# Supplementary material for: Microemulsions: An Encapsulation Strategy to Increase the Thermal Stability of D-limonene
Source: Pharmaceutics. 2023 Nov 1;15(11):2564. doi: 10.3390/pharmaceutics15112564 (PMC10674340; doi:10.3390/pharmaceutics15112564)
Supplement: Supplementary file 1 [file pharmaceutics-15-02564-s001.zip › pharmaceutics-2664275-supplementary.pdf]

Figure S1. Analytical scanning (190 to 450 nm) of mixture Labrasol : water (70:30).

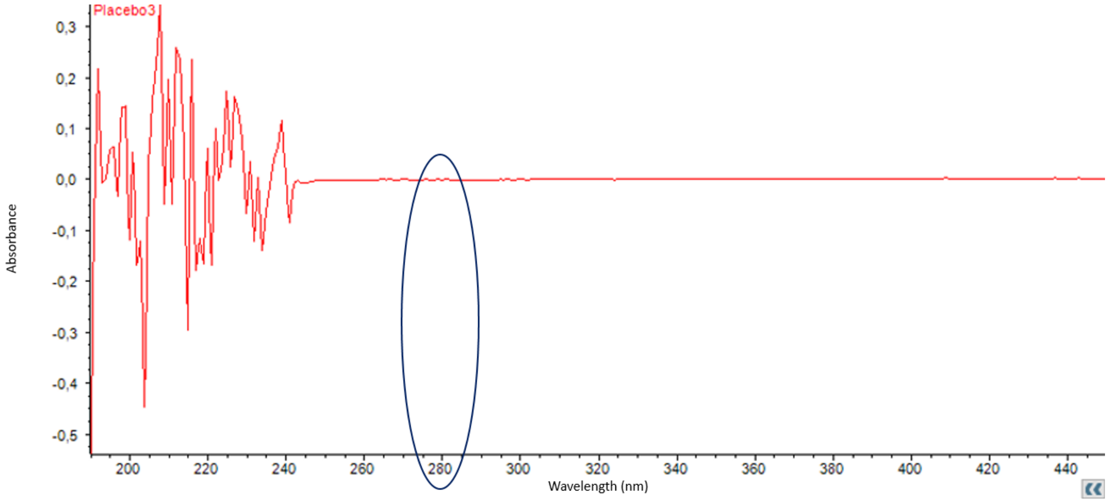

Figure S2. Analytical scanning (190 to 450 nm) of absolute ethanol.

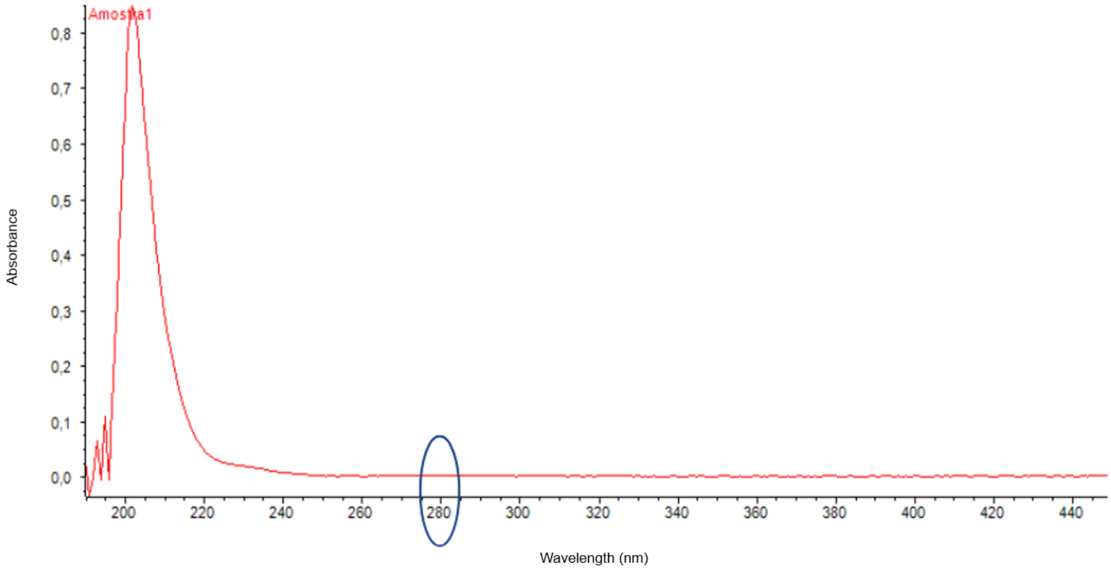

Figure S3. Analytical calibration curve of D-limonene concentration as a function of absorbance.

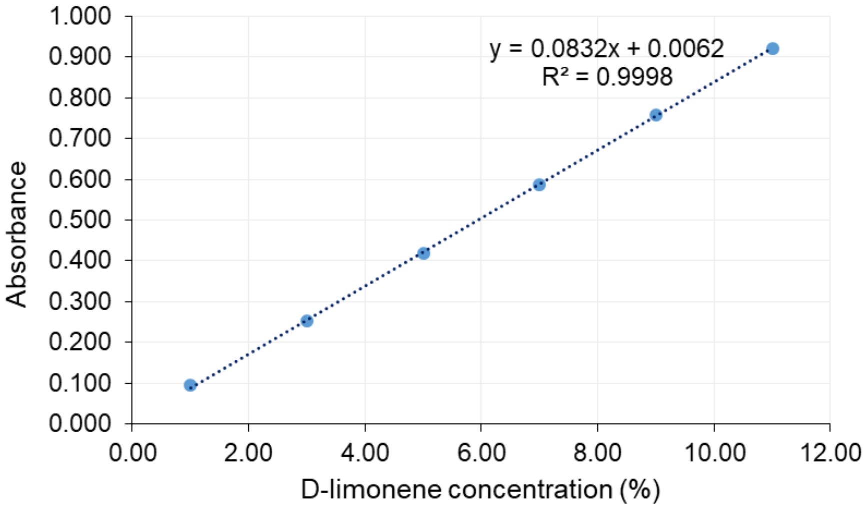

Figure S4. Formulations obtained based on a required HLB of 6.4, using the surfactant mixture of 27.27% Labrasol® and 72.73% Span 80.

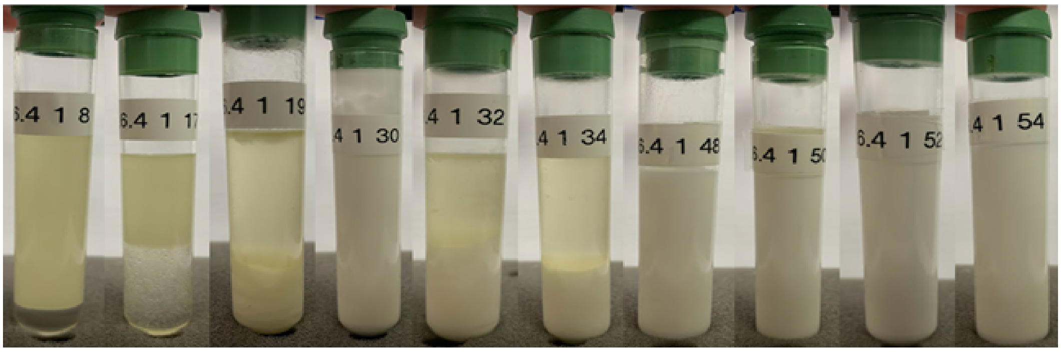

Figure S5. Formulations obtained based on a required HLB of 6.4, using the surfactant mixture of 45.1% Labrasol® + 54.9% Span 85.

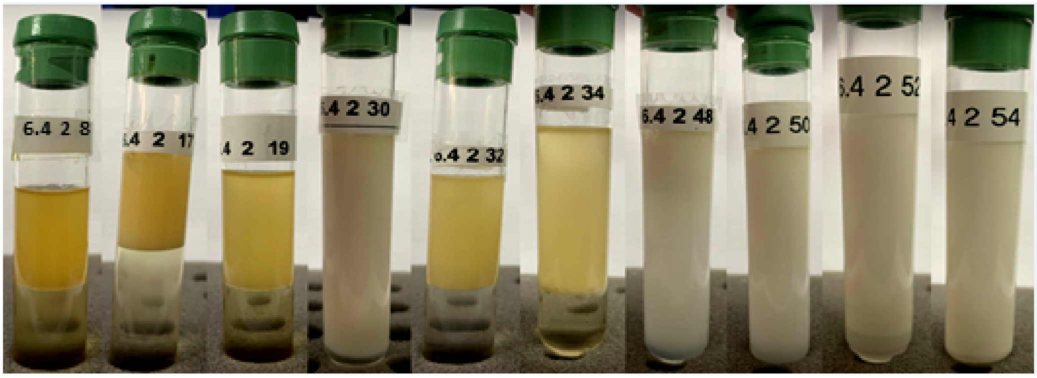

Figure S6. Formulations obtained based on a required HLB of 6.4, using the surfactant mixture of 19.81% Tween 60 + 80.19% Span 80.

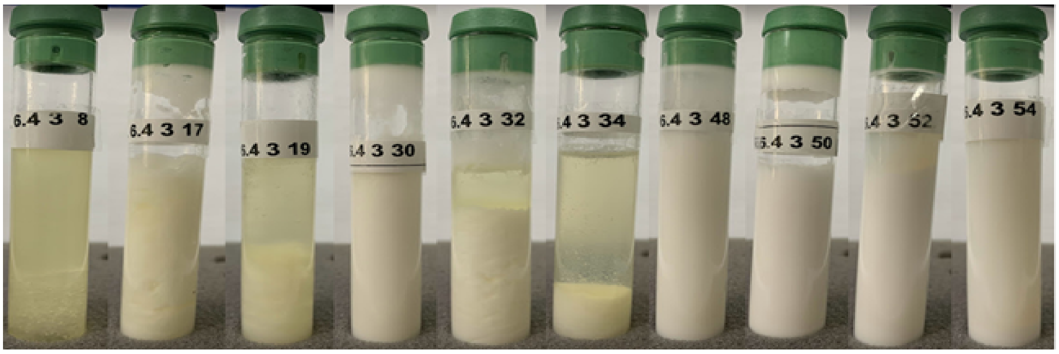

Figure S7. Formulations obtained based on a required HLB of 6.4, using the surfactant mixture of 35.11% Tween 60 and 64.89% Span 85.

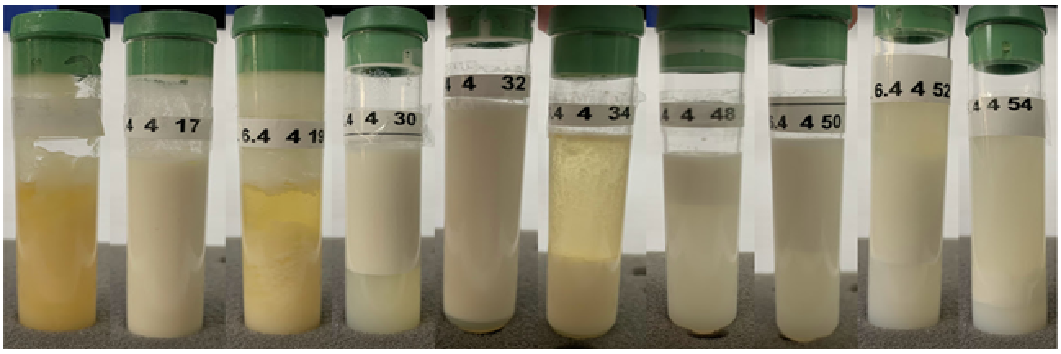

Figure S8. Formulations obtained based on a required HLB of 8.7, using a surfactant mixture of 37.74% Labrasol® and 62.26% Span 40.

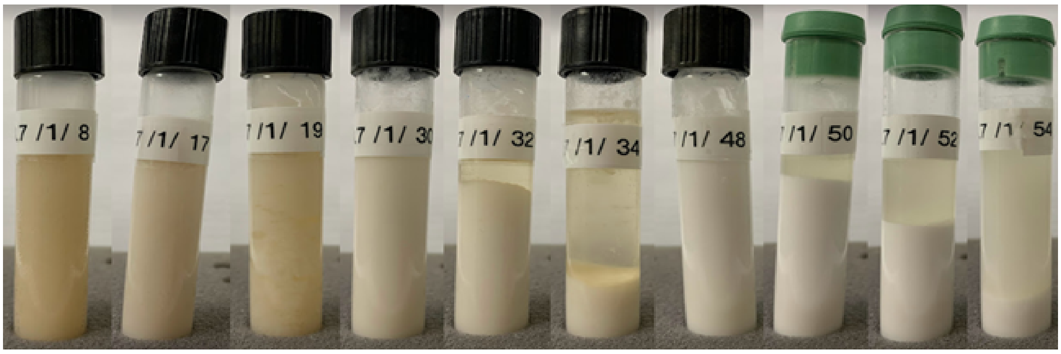

Figure S9. Formulations obtained based on a required HLB of 8.7, using a surfactant mixture of 57.14% Labrasol® and 42.86% Span 80.

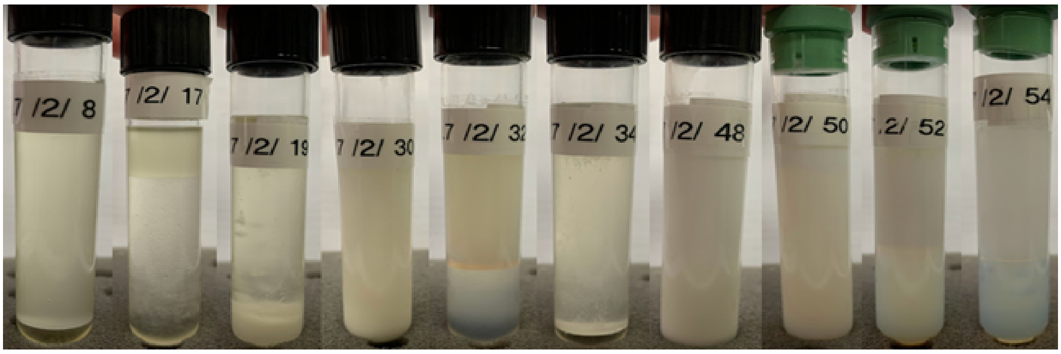

Figure S10. Formulations obtained based on a required HLB of 8.7, using a surfactant mixture of 24.39% Tween 60 and 75.61% Span 40.

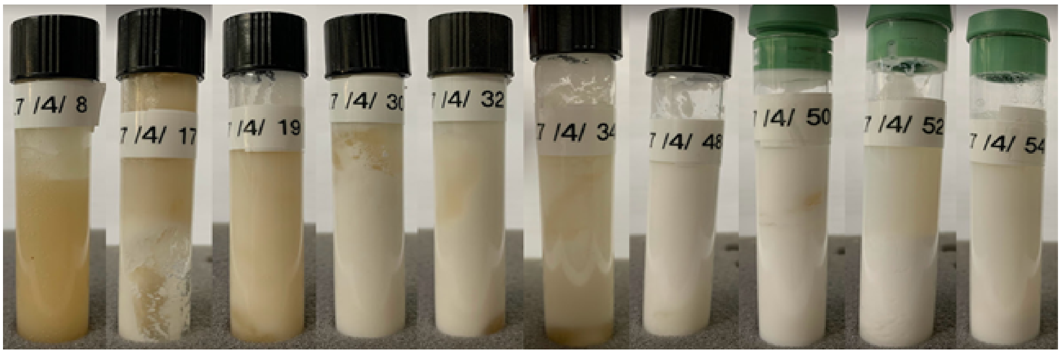

Figure S11. Formulations obtained based on a HLB required equal to 8.7, using a surfactant mixture of 41.51% Tween 60 + 58.49% Span 80.

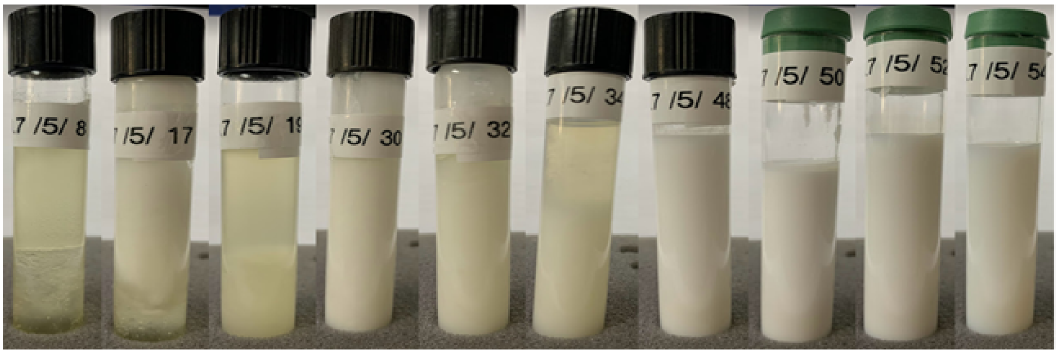

Figure S12. Formulations obtained based on a required HLB of 8.7, using a surfactant mixture of 52.67% Tween 60 and 47.33% Span 85.

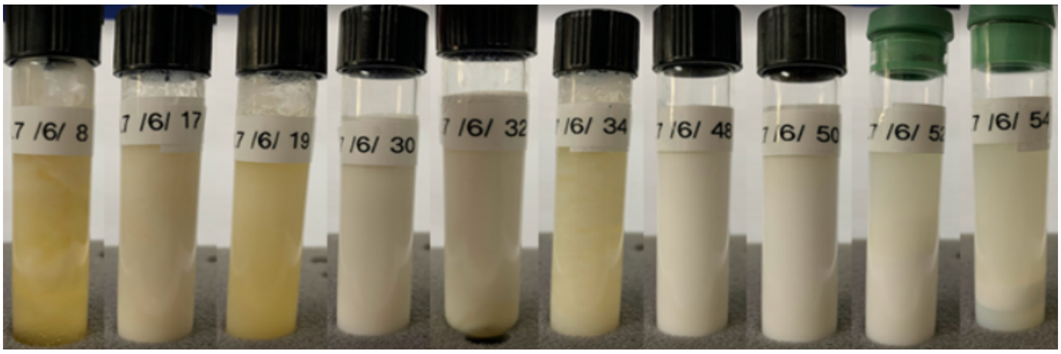

Figure S13. Formulations obtained using the surfactant mixture for an HLB requirement of 12, with reference to 72.64% Tween 60 + 27.36% Span 80.

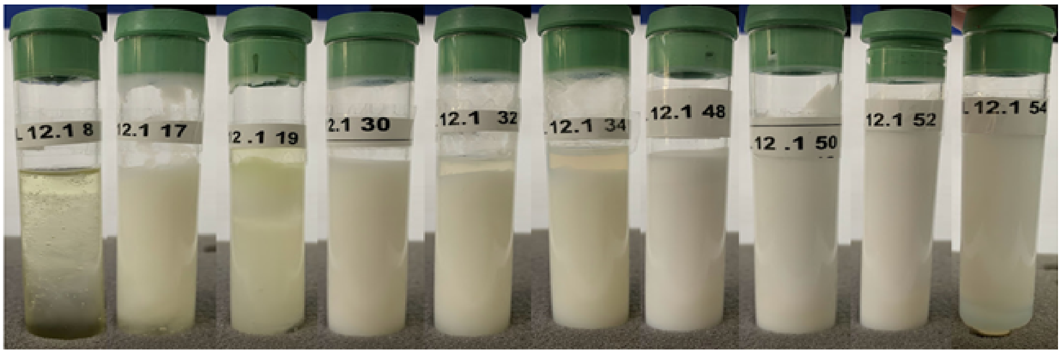

Figure S14. Formulations obtained based on a HLB requirement of 12, using a surfactant mixture of 77.86% Tween 60 and 22.14% Span 85.

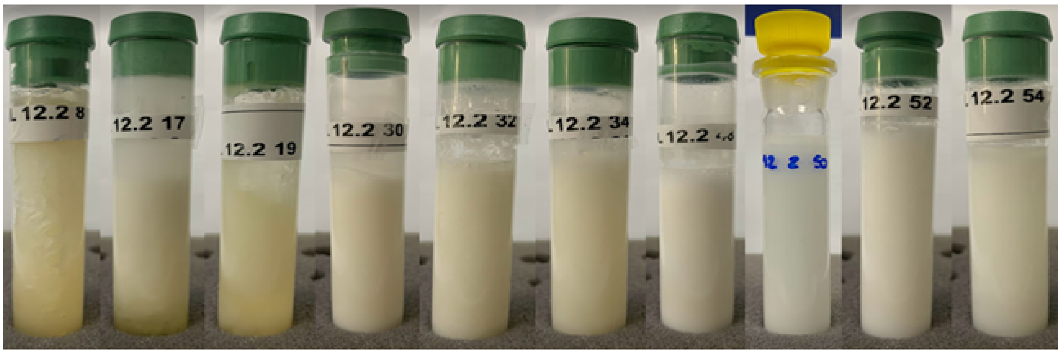

Figure S15. Formulation Obtained via Titration A, derived from a mixture of 55% D-limonene and 45% Labrasol.

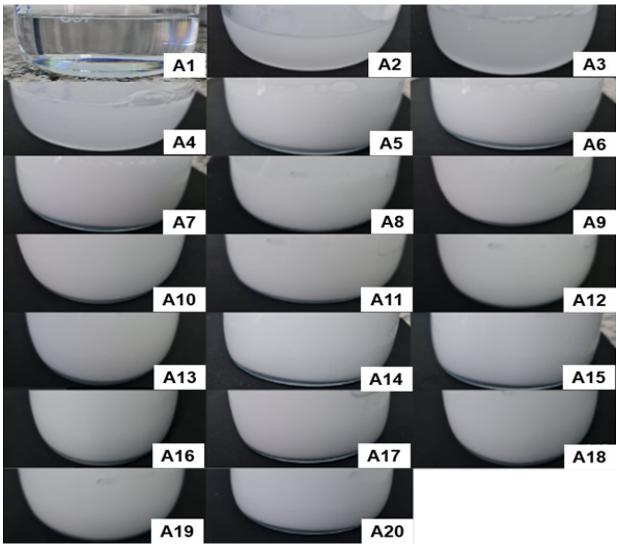

Figure S16. Resulting formulation from titration B, derived from a mixture of 40% D-limonene and 60% Labrasol.

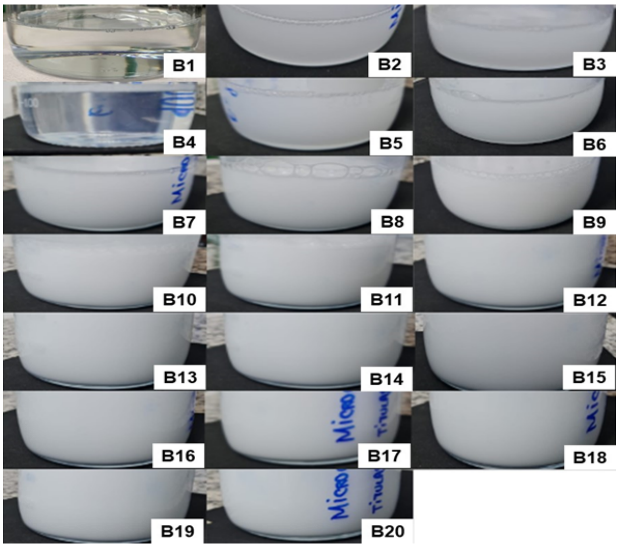

Figure S17. Resulting formulation from titration C, derived from a mixture of 30% D-limonene and 70% Labrasol.

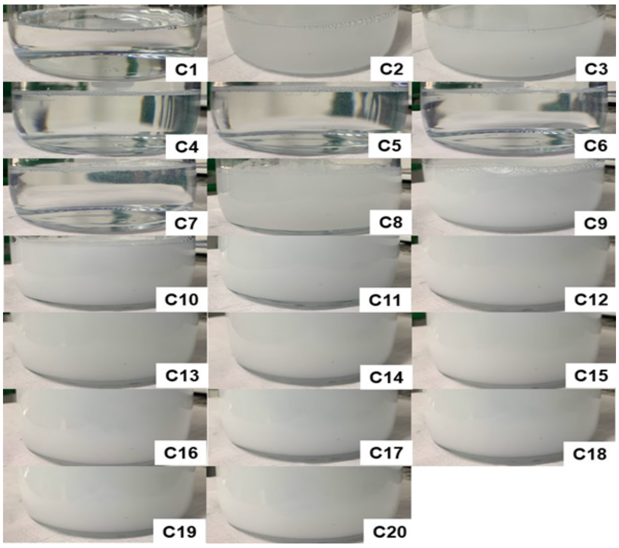

Figure S18. Resulting formulation from titration D, derived from a mixture of 20% D-limonene and 80% Labrasol.

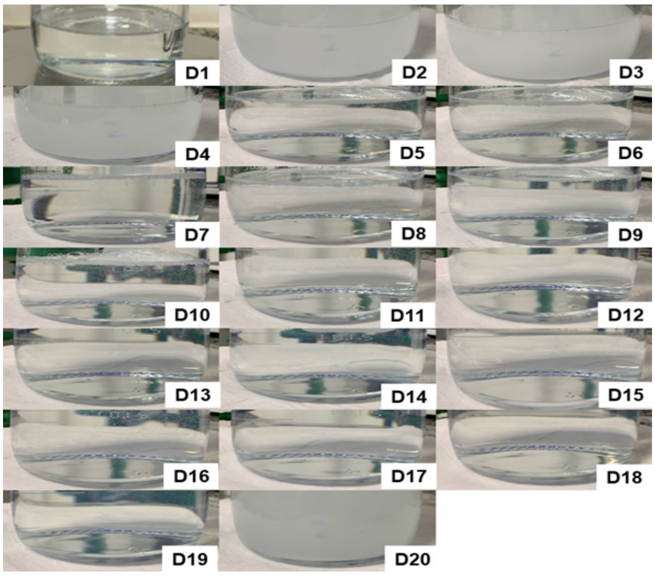

Table S1. Theoretical concentration of D-limonene, practical concentration of D-limonene, mean, standard deviation (n=3), and average content of D-limonene.

Formulation	Theoretical concentration of d-limonene (%)	Mean Concentration (%)	Standard deviation	Average content (%)
B4	30.800	31.000	0.292	100.649
C4	23.100	23.244	0.028	100.622
C5	21.400	21.304	0.028	99.554
C6	20.000	21.000	0.000	105.000
C7	18.800	18.917	0.056	100.621
D5	14.300	14.301	0.121	100.009
D6	13.300	13.660	0.073	102.709
D7	12.500	12.747	0.121	101.974
D8	11.800	11.897	0.028	100.826
D9	11.100	11.272	0.073	101.553
D10	10.500	10.923	0.036	104.029
D11	10.000	10.430	0.024	104.303
D12	9.500	10.046	0.024	105.744
D13	9.100	9.625	0.087	105.769
D14	8.700	9.104	0.060	104.646
D15	8.300	8.535	0.069	102.834
D16	8.000	8.267	0.062	103.355
D17	7.700	7.742	0.046	100.545
D18	7.400	7.429	0.037	100.398
D19	7.100	7.173	0.024	101.029
F17	10.000	10.647	0.028	106.474
F19	30.000	31.417	0.309	104.722

Table S2. Results of the oxidative stability of D-limonene based on maximum pressure, final pressure, and induction period.

Sample	Maximum pressure (kPa)	Final pressure (kPa)	Induction period (min)
D-limonene	513.50	461.70	4.88
B4	688.60	619.00	19.18
B4 placebo	695.60	625.90	61.28
C4	703.60	632.90	22.67
C4 placebo	694.60	624.90	36.83
C5	703.60	632.90	22.18
C5 placebo	703.60	632.90	41.17
C6	697.60	626.90	22.83
C6 placebo	666.70	600.10	47.57
C7	697.60	626.90	22.57
C7 placebo	701.60	630.90	49.93
D5	698.60	627.90	29.48
D5 placebo	694.60	624.90	36.02
D6	697.60	626.90	29.55
D6 placebo	696.60	626.90	46.10
D7	700.60	629.90	34.17
D7 placebo	705.60	634.90	57.37
D8	705.60	634.90	36.58
D8 placebo	704.60	633.90	74.53
D9	687.60	618.00	37.65
D9 placebo	709.50	637.90	58.17
D10	684.70	616.00	39.30
D10 placebo	703.60	632.90	80.98
D11	696.60	626.90	40.33
D11 placebo	704.60	633.90	67.88
F17	656.80	591.10	19.43
F17 placebo	636.70	572.00	63.02
F19	673.70	606.00	29.83
F19 placebo	632.70	569.10	66.67
